# Understanding Heroin Overdose: A Study of the Acute Respiratory Depressant Effects of Injected Pharmaceutical Heroin

**DOI:** 10.1371/journal.pone.0140995

**Published:** 2015-10-23

**Authors:** Caroline J. Jolley, James Bell, Gerrard F. Rafferty, John Moxham, John Strang

**Affiliations:** 1 Division of Asthma, Allergy and Lung Biology, Faculty of Life Sciences and Medicine, King’s College London, King’s Health Partners, Denmark Hill, London, United Kingdom; 2 National Addiction Centre, Institute of Psychiatry, Psychology and Neuroscience, King’s College London, King’s Health Partners, Denmark Hill, London, United Kingdom; 3 Addictions Services, South London & Maudsley NHS Foundation Trust, King’s Health Partners, Denmark Hill, London, United Kingdom; Medical University of Vienna, AUSTRIA

## Abstract

Opioids are respiratory depressants and heroin/opioid overdose is a major contributor to the excess mortality of heroin addicts. The individual and situational variability of respiratory depression caused by intravenous heroin is poorly understood. This study used advanced respiratory monitoring to follow the time course and severity of acute opioid-induced respiratory depression. 10 patients (9/10 with chronic airflow obstruction) undergoing supervised injectable opioid treatment for heroin addiction received their usual prescribed dose of injectable opioid (diamorphine or methadone) (IOT), and their usual prescribed dose of oral opioid (methadone or sustained release oral morphine) after 30 minutes. The main outcome measures were pulse oximetry (SpO_2_%), end-tidal CO_2_% (ETCO_2_%) and neural respiratory drive (NRD) (quantified using parasternal intercostal muscle electromyography). Significant respiratory depression was defined as absence of inspiratory airflow >10s, SpO_2_% < 90% for >10s and ETCO_2_% per breath >6.5%. Increases in ETCO_2_% indicated significant respiratory depression following IOT in 8/10 patients at 30 minutes. In contrast, SpO_2_% indicated significant respiratory depression in only 4/10 patients, with small absolute changes in SpO_2_% at 30 minutes. A decline in NRD from baseline to 30 minutes post IOT was also observed, but was not statistically significant. Baseline NRD and opioid-induced drop in SpO_2_% were inversely related. We conclude that significant acute respiratory depression is commonly induced by opioid drugs prescribed to treat opioid addiction. Hypoventilation is reliably detected by capnography, but not by SpO_2_% alone. Chronic suppression of NRD in the presence of underlying lung disease may be a risk factor for acute opioid-induced respiratory depression.

## Introduction

Globally, heroin/opiate overdose is a major cause of death amongst young adults. As the World Health Organization [1] observes: *“Opioids are potent respiratory depressants, and overdose is a leading cause of death among people who use them. Worldwide, an estimated 69,000 people die from opioid overdose each year.”* Opiates loom large in statistics on drug-related deaths. In England and Wales, more than 1,700 deaths registered in 2014 (53% of all deaths from drug poisoning) involved an opiate drug [2]. However, given the very nature of the event, most research has either been epidemiological (e.g [3, 4]), toxicological (e.g [5]) or post-mortem (e.g [6]). With the recent establishment of supervised injectable heroin maintenance clinics (see description in Lancet 2010 [7] and recent meta-analysis [8]), an opportunity exists to study physiological responses to intravenous and intramuscular administration of extremely high dose diamorphine (e.g. 50–200mg) in a clinical context, but with the addition of comprehensive monitoring of acute changes in respiratory function.

Evidence from international trials suggests that injectable opioid treatment (IOT) provided in supervised clinics can be an effective intervention for patients who fail to respond to oral methadone treatment for heroin addiction. These trials have demonstrated that in methadone non-responders, injectable treatment can markedly reduce use of street drugs and contribute to improvements in health and wellbeing. By guaranteeing prompt treatment in the event of acute respiratory depression, directly supervised opioid treatments also offer additional security compared to continued illicit opioid use on the street. However, injectable trials have consistently reported a higher incidence of serious adverse events—notably, respiratory depression—associated with injectable compared to oral treatment [7, 9–13]. The risk of respiratory depression is greatest if patients have concomitantly taken benzodiazepines or alcohol, or during induction while tolerance to opioids is being increased. The great majority of adverse events occur within a few minutes of injection, leading some clinicians to suggest that all patients treated in injectable clinics should be monitored for 15 minutes after every injection [14]. Standard practice involves monitoring for hypoventilation using pulse oximetry. It is unknown, however, whether other approaches to monitoring, such as transcutaneous or end-tidal CO_2_ monitoring, or more direct measures of neural respiratory drive (NRD) such as respiratory muscle electromyography [15–18] provide a more sensitive and earlier indicator of respiratory depression in these patients.

The aim of this study was to investigate the time course and severity of respiratory depression following acute administration of injected and oral opioids in opioid-tolerant subjects. Specifically, we aimed to investigate the use of a combination of pulse oximetry, end-tidal CO_2_ monitoring, and parasternal intercostal muscle electromyogram recordings (EMGpara) to detect acute reductions in neural respiratory drive (NRD) to determine the added value of advanced physiological monitoring over pulse oximetry alone.

## Methods

### Ethical approval

The study was approved by the Clinical Governance Committee, South London and Maudsley Addictions Clinical Academic group, and was conducted according to the principles expressed in the Declaration of Helsinki. Consent was given orally and recorded in the patient’s clinical notes, as approved by the Clinical Governance Committee.

### Inclusion and exclusion criteria

#### Inclusion criteria

1. Receiving injectable opioid treatment for heroin addiction by South London and Maudsley NHS Foundation Trust, on a stable dose regime for at least 2 weeks;
2. Age >/ = 18 years;
3. Capacity to provide informed consent.

#### Exclusion criteria

1. Acute drug or alcohol intoxication or delirium tremens;
2. Inability to reliably perform physiological tests of respiratory function;
3. Concomitant benzodiazepine use.

### Participants

10 patients took part in the study. Participants’ age, height, weight and body mass index (BMI) were documented. Spirometry (forced expiratory volume in 1 second (FEV_1_) and slow vital capacity (VC)) were measured and reported as FEV_1_% predicted, VC% predicted and FEV_1_%VC.

### Protocol

Each participant attended the respiratory unit and had monitoring equipment fitted. Once comfortable, and having had baseline observations performed, participants received their prescribed dose of injectable opioid (at t = 0 minutes). Each participant was then monitored over a period of 150 minutes after intravenous and intramuscular high-dose diamorphine (pharmaceutical heroin), with the dose having previously been determined in the context of their addiction treatment. EMGpara and pulse oximetry (SpO_2_%) were monitored continuously over the study duration. At 3 minutes prior to administration of the injectable opioid, and then at 3, 8, 15, 30, 60, 105 and 150 minutes each participant was asked to rate their subjective feeling of drug effect using a visual analogue scale, and one-minute averages of end tidal CO_2_% (ETCO_2_%) and airflow were recorded. At these times pupil size was also recorded, and a staff rating of level of consciousness and intoxication were also documented.

### Measurements

All physiological signals were acquired, digitised and analysed using a Powerlab analog-to-digital converter (ADInstruments Pty Ltd, Castle Hill, Australia) on a laptop computer (Apple Computer Inc, Cupertino, CA USA) running LabChart Pro software (LabChart 7 Pro version 7.3.7, ADInstruments Pty Ltd, Castle Hill, Australia). Data were stored for offline analysis using LabChart 7 Pro.

#### Parasternal intercostal muscle electromyogram recordings (EMGpara)

EMGpara was recorded using bipolar surface electrodes (Kendall Arbo^®^, Tyco Healthcare^®^, Neustadt.Germany) applied bilaterally in the second intercostal space, 3cm from the midline, as previously described [19]. EMGpara signals were amplified and band-pass filtered between 10 Hz and 3 kHz (Biomedical amplifier Pclab-3808, Guangzhou Yinghui Medical), and acquired and digitized at a sampling frequency of 2kHz with additional digital band-pass filtering between 20Hz and 1kHz for EMG analysis. Peak root mean square per breath was calculated and expressed as a percentage of the maximum EMG (EMGpara%max) obtained during three maximal volitional manoeuvres: inspiration to total lung capacity, maximal static inspiratory pressure manoeuvre and a maximal sniff manoeuvre [17]. To incorporate the combined effect of opioids on neural respiratory drive (NRD) per breath and respiratory rate, the “EMGpara%index” was calculated as the product of EMGpara%max and respiratory rate (V_f_).

#### Ventilation

Airflow was measured through a mouthpiece connected in series to a Fleisch pneumotachograph with a noseclip in place. Tidal volume per breath (V_T_) was calculated offline as the integral of the flow signal. Minute ventilation (V_E_) was calculated as the product of the average V_T_ and V_f_.

#### SpO_2_% and ETCO_2_%

SpO_2_% was recorded by pulse oximetry (Ohmeda Biox 3700). SpO_2_% was consistently increased upon breathing through the pneumotachograph during the intermittent measures of ventilation. Hence SpO_2_% for each timepoint was reported as the average of data over the 30 second period immediately preceding the time at which the pneumotachograph was introduced.

ETCO_2_% was measured as the peak %CO_2_ per expired breath using a capnograph (GA-200, iworx, New Hampshire, US) which sampled continuously from using a fine bore catheter pneumotachograph.

#### Indicators of significant respiratory depression

Recordings were examined for evidence of the following indices of significant respiratory depression: absence of inspiratory airflow for more than 10 seconds, SpO_2_ < 90% for more than 10 seconds and ETCO_2_% per breath exceeding 6.5%. Participants were stimulated with verbal and/or painful stimuli if apnoea persisted > 15 seconds.

#### Subjective drug effect

Staff rating of intoxication was measured on a visual analogue scale:

Rating of intoxication 0 = no effect, 5 = maximal effect

Staff level of consciousness was assessed using a numerical rating scale:

1 = normal, 2 = visibly affected but alert, 3 = drowsy but responds to verbal stimuli, 4 = no response to verbal stimuli

Patients assessed their drug-related “high” on a visual analogue scale:

Rating of intoxication 0 = no effect, 5 = maximal effect

#### Statistics

Statistical analysis was performed using GraphPad Prism 5 for Windows v5.00 (GraphPad Software, Inc). Data are presented as the median and interquartile range unless otherwise stated. Differences between physiological variables between baseline and successive timepoints after drug administration were assessed using 1 way ANOVA (Friedman test), with post hoc analysis using Dunn’s Multiple Comparison Test. Statistical significance levels were set at p<0.05.

## Results

Demographic and anthropometric data, lung function and baseline levels of EMGpara%max and EMGpara% index are shown in Table 1.

**Table 1:** Demographic and anthropometric data, spirometry and EMGpara data of the 10 participants.

	Median	Interquartile range
**Age (years)**	49	42 to 58
**Sex (M/F)**	8/2	
**Height (m)**	1.75	1.62 to 1.81
**Weight (kg)**	65.6	54.8 to 76.4
**BMI (kg/m** ^**2**^ **)**	21.6	19.3 to 25.3
**FEV**_**1**_ **(L)**	2.30	1.48 to 3.78
**FEV**_**1**_ **%predicted**	76.0	54.0 to 95.5
**VC (L)**	4.80	2.98 to 5.53
**VC % predicted**	115.0	101.5 to 116.3
**FEV**_**1**_ **/VC%**	58.5	39.6 to 66.1
**EMGpara%max (%)**	8.20	6.53 to 18.42
**EMGpara%index (a.u.)**	109.5	69.5 to 185.1

Data are presented as the median and interquartile range. M = male, F = female, BMI = body mass index, FEV_1_ = forced expiratory volume in 1 second, VC = vital capacity, EMGpara%max = parasternal electromyogram activity per breath as a proportion of maximum, EMGpara%index = EMGpara%max*respiratory rate, a.u. = arbitrary units.

Nine of the 10 participants had chronic obstructive pulmonary disease (COPD) by spirometric and clinical criteria. Only one participant had previously been diagnosed with COPD and had been prescribed inhaled short-acting beta-2-agonists. This participant reported infrequent use of inhaled treatment, and had not used inhalers on the day of testing. Seven participants were taking additional prescribed medication as follows: mirtazapine 45mg once daily only (2 patients); fluoxetine 20mg once daily only (2 patients); zopiclone 7.5mg tablet nocte and co-codamol 8mg/500mg tablets as required (1 patient); beta blocker (not specified) and omeprazole (1 patient); amitriptyline 75mg once daily and fluoxetine 20mg once daily (1 patient).

Periodic breathing was evident from the SpO_2_%, airflow and EMGpara traces before administration of study drugs in eight of the ten participants.

All participants completed 30 minutes of recording after administration of an injectable opioid (three by intravenous injection; seven by intramuscular injection), and nine of the ten subjects took an oral opioid. The majority of patients chose to finish the study before the intended 150 minutes, citing other time commitments as the reason for early departure. The median time spent undergoing recordings after administration of an oral opioid was 60 minutes (range 50 to 120 minutes), with only one participant completing the study to the planned 150 minutes post injectable opioid.

### Summary of main respiratory effects

**Fig 1:**
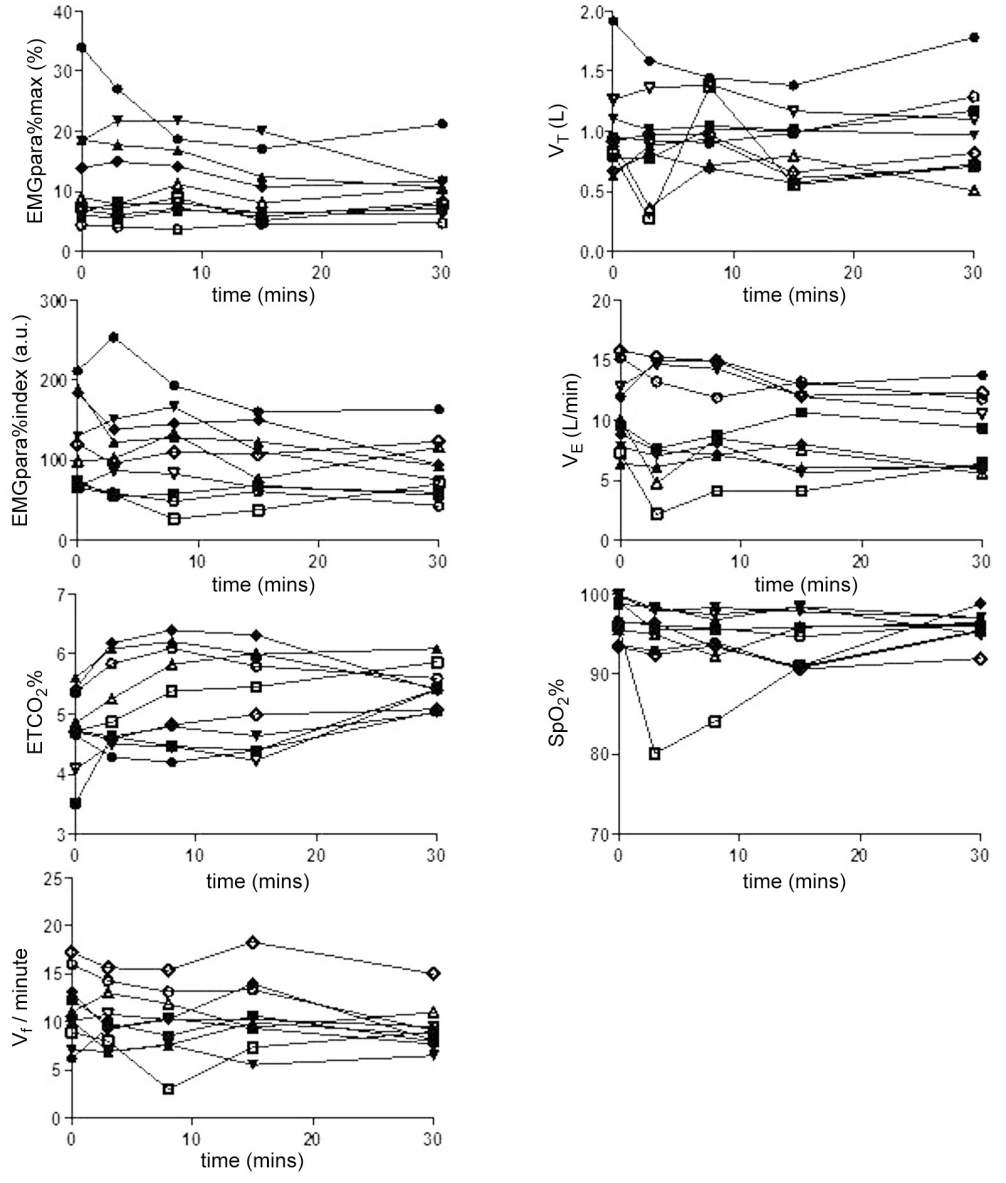
Physiological effects of injectable opioids on individual participants. One-minute averages of physiological data for each of the 10 individuals at baseline (0 minutes) and 3 minutes, 8 minutes, 15 minutes and 30 minutes after injectable opioid administration. V_T_ = tidal volume, V_E_ = minute ventilation, V_f_ = respiratory rate, SpO_2_% = oxygen saturation measurement determined by pulse oximetry, ETCO_2_% = end tidal breath carbon dioxide concentration (%).

The impact of injection (given at T0) and of oral methadone (administered 30 minutes later) is shown in Fig 1 and Table 2.

**Table 2:** Dosage, routes of administration, episodes of respiratory depression following injected and oral opioid medication for each participant.

ID	Drug T0	Resp pause >10s	SpO_2_ <90% for >10s	ETCO_2_ > 6.5%	Oral opioid T30	Resp pause >10s	SpO_2_ <90% for >10s	ETCO_2_ > 6.5%
1	diamorphine 50mg IV				methadone 80mg		✓	
2	diamorphine 60mg IV				None	a	a	a
3	diamorphine 90mg IM	✓		✓	methadone 50mg	✓		✓
4	diamorphine 100mg IM	✓			SROM 700mg			
5	diamorphine 200mg IM			✓	methadone 100mg			✓
6	diamorphine 160mg IM	✓		✓	methadone 80mg			✓
7	diamorphine 100mg IV	✓	✓	✓	SROM 600mg	✓	✓	✓
8	diamorphine 200mg IM		✓	✓	methadone 100mg			
9	methadone 100mg IM				methadone 100mg			
10	diamorphine 160mg IM		✓	✓	methadone 100mg			

^✓^ indicates the occurrence of the corresponding adverse event.

^a^ indicates data not collected.

ID = patient identification number. T0 = baseline. T30 = 30 minutes after injected opioid. “0–30” indicates the time period between T0 and T30. “30–60” indicates the time period between T30 and 60 minutes post injected opioid. Resp pause > 10s = absence of inspiratory airflow for more than 10s. IM = intramuscular injection. IV = intravenous injection. SROM = sustained release oral morphine.

**Fig 2:**
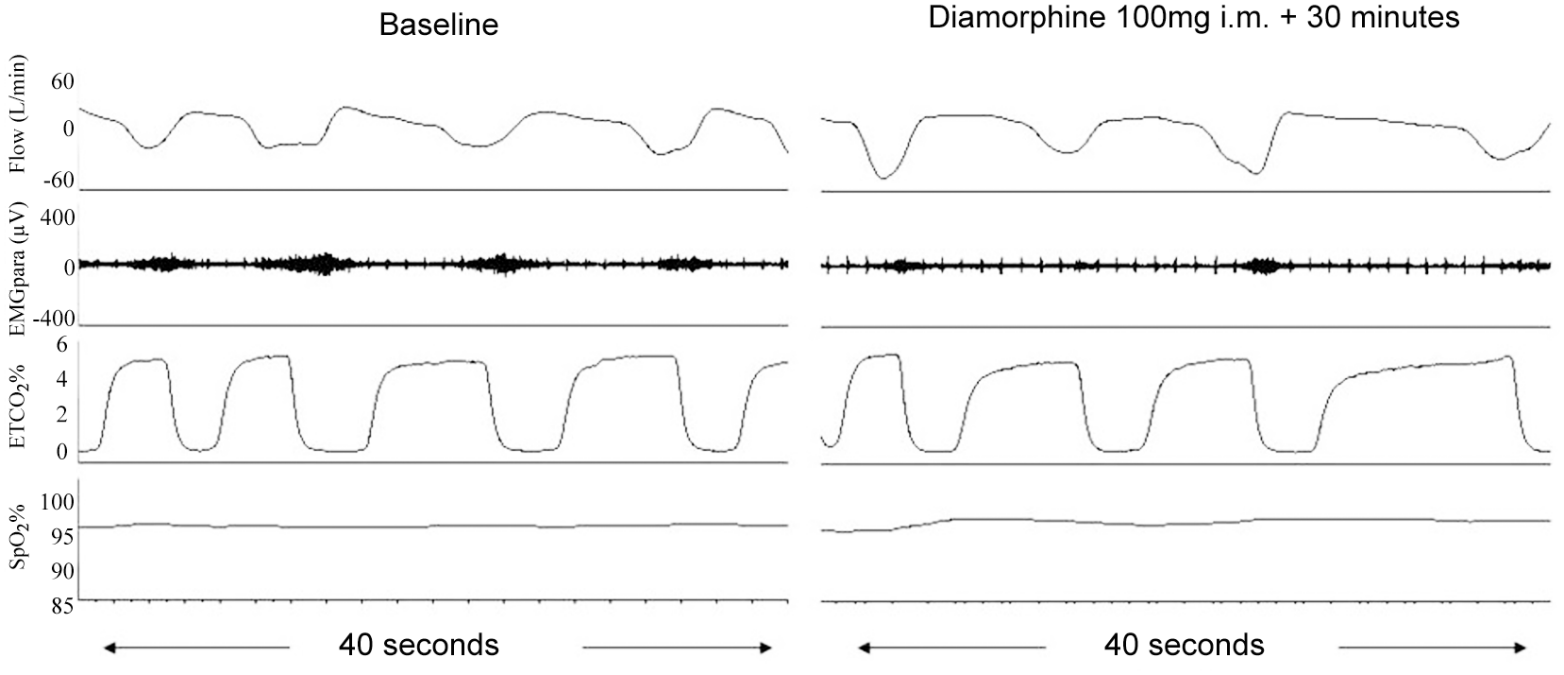
Airflow, parasternal electromyogram activity (EMGpara), ETCO_2_% and SpO_2_% in an individual subject (#4) at baseline and at 30 minutes after intramuscular diamorphine injection. Airflow, parasternal electromyogram activity (EMGpara), ETCO_2_% and SpO_2_% in an individual subject (#4) at baseline and at 30 minutes after intramuscular administration of 100mg diamorphine. Note substantial reductions in EMGpara activity per breath despite relatively little change in airflow, respiratory rate, ETCO_2_% and SpO_2_%.

After injectable rather than oral opioids, most patients (eight) were observed to experience at least one of the indicators of respiratory depression for which monitoring was performed (Table 2). In six of the participants, at least two of these indicators were seen. Over the duration of the study, the median (range) nadir of SpO_2_% was 88.3% (73.6 to 92.6) and the median (range) peak ETCO_2_% per breath was 6.9% (5.2 to 7.8). Oxygen desaturation was mostly periodic, but the average SpO_2_% was significantly lower at 15 minutes post injectable opioid (baseline SpO_2_ 96.5 (95.1 to 99.2)% to 95.3 (90.9 to 98.4)% at 15 minutes, p = 0.03). However, SpO_2_% frequently remained close to baseline (pre injectable opioid) levels despite significant reductions in neural respiratory drive as indicated by a fall in EMGpara activity (Fig 2).

**Fig 3:**
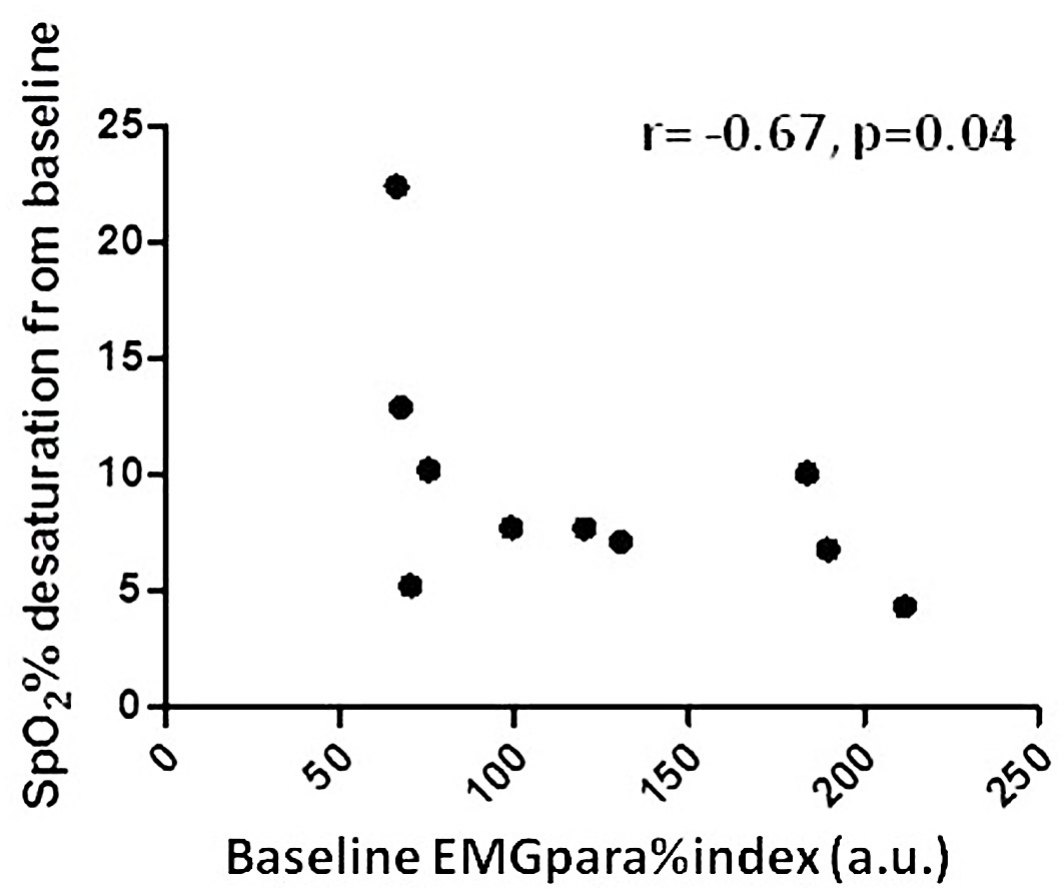
The relationship between baseline EMGpara%index and the magnitude of the maximum change in SpO_2_% from baseline. Data shown are for each individual participant. r = Spearman coefficient; a.u. = arbitrary units.

There was a significant inverse relationship between EMGpara%index at baseline and the magnitude of the opioid-induced drop in SpO_2_ from baseline (Spearman r = -0.67, p = 0.04), i.e. opioid-induced oxygen desaturation was greater in participants with lower levels of neural respiratory drive at baseline (Fig 3).

### Acute effects of injectable opioids on neural respiratory drive and ventilation

EMGpara%index declined at a variable rate from baseline (baseline EMGpara%index 109.5 (69.5 to 185.1) arbitrary units (a.u.) to 84.3 (59.2 to 118.1) a.u. at 30 minutes, p = 0.12, accompanied by significant increases in ETCO_2_% (median (IQR) baseline ETCO_2_% 4.7 (4.5 to 5.4)% to 5.4 (5.1 to 5.7)% at 30 minutes, p = 0.01) (Fig 1). In participants with baseline levels of EMGpara%max above the normal range (participants #1, #3 and #4 with levels of EMGpara%max of 34.0%. 18.7% and 18.4% respectively), reductions in EMGpara activity following injectable opioid were proportionally greater than reductions in ventilation (Fig 2).

### Acute effects of oral opioids on neural respiratory drive and ventilation

Nine patients received an oral opioid. The median (IQR) maximum one-minute average of ETCO_2_% recorded after oral opioid administration was 5.8 (4.8 to 6.2)%. The minimum one-minute averages of SpO_2_%, V_E_, EMGpara%max and EMGpara%index were 94.7 (92.9 to 95.9)%, 9.0 (7.5 to 13.4) L/min, 9.3 (6.6 to 13,3)% and 108.7 (67.4 to 117.3) a.u. respectively. Thus relatively little absolute change in respiratory variables was observed after oral opioid administration above any residual effect of injectable opioid after 30 minutes, although reductions in one-minute averages of EMGpara%index were observed in four participants.

### Participant- and observer-reported effects of opioids

Significant changes compared to baseline (t = 0) were reported in observer-reported pupil size and participant-reported subjective “high” 8-minutes after injected opioids. Changes reported after oral opioid administration were not significant. There were no statistically significant changes in level of consciousness or intoxication following injected or oral opioid administration (Table 3).

**Table 3:** Participant-rated drug-related “high”, and observer-rated responses to injected and oral opioids.

	Minutes post injected opioid					Post oral opioid
	0	3	8	15	30	>60 mins
**Pupil size (mm)**	4.0 (3.0 to 7.0)	3.0 (2.0 to 4.0)	2.5 (2.0 to 3.0)*	2.75 (2.0 to 3.0)*	3.0 (2.0 to 3.25)	3 (2.25 to 3.0)
**Consciousness**	1.0 (1.0 to 1.0)	1.0 (1.0 to 1.25)	1.0 (1.0 to 1.25)	1.0 (1.0 to 2.25)	1.0 (1.0 to 2.0)	1 (1.0 to 1.0)
**Subjective high**	0.0 (0.0 to 0.0)	1.5 (0.0 to 2.63)	2.0 (1.0 to 2.25)*	2.0 (1.0 to 2.25)*	2.0 (1.0 to 2.25)*	1.0 (0.0 to 2.0)
**Intoxication (observer-rated)**	0.0 (0.0 to 0.0)	1.0 (0.0 to 2.25)	1.0 (0.0 to 3.25)	1.5 (0.0 to 3.25)	2.0 (0.75 to 4.0)	1.0 (0.0 to 2.5)

Higher numbers on scales for consciousness, subjective high and level of intoxication indicate greater drug-related effects (refer to Methods section for full details).

* indicates a statistically significant change compared to baseline (t = 0), at the 0.05 level

## Discussion

### Principal findings

Significant respiratory suppression was found to occur commonly in the acute period following administration of injectable and oral opioid drugs in opioid-tolerant addicts. Our advanced respiratory monitoring protocol, in particular a combination of pulse oximetry and capnography, was able to detect changes that would have been under-recognised by standard clinical monitoring protocols using pulse oximetry alone. Subjective observer ratings of drug effect were found to be unreliable markers of physiological effect. We also observed an inverse relationship between baseline EMGpara%index and opioid-induced hypoxaemia.

### Strengths and weaknesses of the study

The main strength of the study is that it is the first to document the degree of acute opioid-induced respiratory depression induced in heroin addicts using capnography, and respiratory muscle electromyography which is a quantifiable and more direct measure of NRD than standard pulse oximetry alone. One weakness of the study is the small relatively sample size. However, this is a challenging group of patients on whom to carry out detailed physiological studies, with a small potential cohort of around 40 patients enrolled in such clinics at one time. We were unable to include a group of patients without chronic respiratory disease, which would have provided further information to better define the profile of patients at higher risk of respiratory depression when in treatment. This should be a focus of future studies. Patients awaiting their scheduled injection of diamorphine tend to be in an aroused state, and although every attempt was made to relax participants as much as possible, a tendency to hyperventilation was evident in some at baseline. Post-injection, although participants were encouraged to sit as quietly as possible for the measurements, this was not consistently adhered to and, it was not possible to analyse blocks of data that were contaminated by patients talking or moving. Monitoring ETCO_2_% involves a degree of measurement artefact, as the stimulation of having a mouthpiece in place tended to stimulate breathing [20]. Indeed, the inconvenience of the mouthpiece made it impossible to monitor ETCO_2_ continuously over a number of hours. End-tidal CO_2_ measurements also often underestimate true arterial CO_2_ in patients with chronic airflow obstruction and emphysema, reflecting the ventilation-perfusion abnormalities associated with the underlying lung disorder. However the use of ETCO_2_% measurement did allow monitoring of acute changes in CO_2_ on a breath-by-breath basis. Although transcutaneous monitoring of pCO_2_ would have allowed continuous monitoring to be undertaken, this would have been at the expense of a slower response time. Finally, patients did not routinely undergo urine toxicology screening prior to testing to verify that participants were abstinent from additional non-prescribed/illicit drugs that could alter respiratory drive, for example benzodiazepines, cocaine and amphetamines.

### Comparisons with other studies

It is well-known that the μ-opioids diamorphine, morphine and methadone depress respiration, at least in part, through a direct effect on the brainstem respiratory centres [21]. Both acute [22] and chronic [23] opioid use decrease the hypercapnic and hypoxic ventilatory responses in humans. The minimum SpO_2_% observed in the present study of 88.3 (73.6 to 92.6)% is in keeping with the findings of previous studies undertaken within supervised injecting clinics [24–26]. There was evidence of varying degrees of respiratory depression in all participants, particularly after injected diamorphine, and 8/10 participants experienced apnoeic episodes or prolonged episodes of significant hypoxaemia. It was observed that SpO_2_% levels within the “normal” baseline range for an individual did not reliably exclude the presence of opioid-induced reductions in NRD, or increased ETCO_2_% in keeping with opioid-induced hypoventilation. Using airway occlusion pressure at 100ms (P_0.1_) to monitor NRD, acute opioid-induced decreases in NRD have been observed in surgical populations both with [27] and without [28] detectable changes in gas exchange. Previous studies in opioid-naive obstetric and surgical populations, monitoring transcutaneous pCO_2_ [29, 30] or end-tidal CO_2_ [31], have also documented hypercapnia in the presence of normal respiratory rates and oxygen saturation. SpO_2_% remained within the normal range (average SpO_2_% 98%) in healthy volunteers receiving intravenous remifentanyl despite an average 30% decrease in respiratory rate [32]. Furthermore, acute relief of breathlessness by opioids in palliative care can be achieved with a reduction in respiratory rate but minimal change in SpO_2_% or pCO_2_ [33, 34]. There is no experience of the use of more direct measures of NRD to identify episodes of opioid-induced respiratory depression. Work by our group [15, 17, 18, 35, 36] and others [19, 37–39] has shown that quantification of the electromyogram (EMG) of the obligate inspiratory muscles, in particular the diaphragm (EMGdi) and parasternal intercostal muscles (EMGpara), provides a reliable measure of NRD. In patients with respiratory disease, disordered pulmonary mechanics limit translation of NRD to ventilatory output. In such patients, EMGdi and EMGpara provide a more sensitive marker of changes in NRD than respiratory muscle pressure generation or ventilation [17, 38]. Since there is a high prevalence of smoking [40], chronic obstructive pulmonary disease (COPD) and related respiratory disorders [41] in patients undergoing treatment for drug addiction, EMGpara has potential benefits over the use of ventilation-derived indices as a physiological biomarker of NRD in this patient population.

### Explanations and implications of the findings

Pulse oximetry and observer-rated levels of drug effect under-estimate the true respiratory depressant effect of opioids. Clinically-available monitoring systems, comprising of pulse oximetry, respiratory rate and even end-tidal CO_2_, cannot be fully relied upon to detect the extent of acute opioid-induced respiratory depression. Of note, simply speaking to patients who were having prolonged apnoeic spells was enough stimulation to prompt them to resume breathing. Rousing someone to put a clip on their finger, or simply talking to patients post injection, may mask episodes of hypoxia.

Even though this was a small study in a heterogeneous population, including participants on different oral opioids and variable parenteral doses, acute opioid-induced respiratory depression was evident in all study participants to a greater or lesser degree (Fig 1 and Table 2). Thus tolerance to opioids does not seem to fully protect against respiratory depression, which importantly implies that even well-stabilised patients are at risk. Co-administration of additional central nervous system depressants, such as benzodiazepines and alcohol, is a major contributor to heroin-related deaths [42], and would be expected to potentiate the opioid-induced respiratory depression observed here.

Patients with lower levels of NRD at baseline exhibited a greater reduction in SpO_2_ following opiate administration. Levels of EMGpara%max of 8.20 (6.53–18.4) % fall within the range reported in healthy adults [17, 43], below the levels reported in patients with chronic obstructive lung disease [17, 18, 35]. A combination of chronic suppression of NRD by long-term opioids and other CNS depressants, coupled with the progression of lung and other systemic disease, could go some way to explaining the observed higher rate of opioid overdose deaths in older cohorts of opioid addicts [44, 45]. Interestingly, in participants with relatively high levels of EMGpara activity at baseline, in keeping with the presence of COPD and/or other structural lung disease [17, 35], the magnitude of acute decreases in NRD was proportionally greater than reductions in ventilation, SpO_2_ and ETCO_2_%. This can be explained by considering the degree of neuroventilatory uncoupling in these patients. In the presence of altered pulmonary mechanics associated with chronic lung disease, there is significant uncoupling of ventilation from NRD, such that increases in motor efferent command to the respiratory muscles cannot be translated to effective changes in respiratory muscle pressure generation or ventilation. In COPD, increases in NRD, e.g. during exercise, occur with relatively little increase in ventilation or associated physiological parameters after a threshold level of neuroventilatory uncoupling is reached [17, 35, 38, 46]. It is conceivable that a similar phenomenon occurs when NRD is reduced back down the NRD-ventilation relationship curve by opioids, such that reductions in NRD are not accompanied by changes in ventilatory output until a threshold of relative neuroventilatory “recoupling” is achieved.

### Unanswered questions and future research

In this study we have demonstrated that acute opioid-induced respiratory depression in opioid-tolerant addicts can be detected using continuous advanced respiratory monitoring techniques, and that these adverse drug effects are likely to be under-recognised using standard monitoring protocols. It remains unanswered, both within the field of addiction or in general medical settings, whether more intensive respiratory monitoring can reduce the risk of significant opioid-associated respiratory depression. The findings of our study highlight the need for careful physiological studies of the mechanisms of overdose in heroin addiction.

## Declarations

### Transparency declaration

CJJ and JB affirm that the manuscript is an honest, accurate, and transparent account of the study being reported; that no important aspects of the study have been omitted; and that any discrepancies from the study as planned (and, if relevant, registered) have been explained.
