# Correction: Understanding Heroin Overdose: A Study of the Acute Respiratory Depressant Effects of Injected Pharmaceutical Heroin

**DOI:** 10.1371/journal.pone.0143672

**Published:** 2015-11-18

**Authors:** The PLOS ONE Staff

There are errors in the Funding section. The publisher apologizes for these errors. The correct funding information is as follows: Dr. Caroline Jolley is supported by a NIHR Academic Clinical Lectureship in Respiratory Medicine. Professor John Strang is supported by the National Institute for Health Research (NIHR) Biomedical Research Centre for Mental Health at South London and Maudsley NHS Foundation Trust and King's College London. This research received funding support from the National Institute for Health Research (NIHR) Biomedical Research Centre for Mental Health at South London and Maudsley NHS Foundation Trust and King's College London. The funders had no role in study design, data collection and analysis, decision to publish, or preparation of the manuscript.
